# PatternLab for proteomics: a tool for differential shotgun proteomics

**DOI:** 10.1186/1471-2105-9-316

**Published:** 2008-07-21

**Authors:** Paulo C Carvalho, Juliana SG Fischer, Emily I Chen, John R Yates, Valmir C Barbosa

**Affiliations:** 1 Systems Engineering and Computer Science Program, COPPE, Federal University of Rio de Janeiro, Rio de Janeiro, Brazil; 2 Laboratory for Protein Chemistry, Chemistry Institute, and the Rio de Janeiro Proteomic Network, Federal University of Rio de Janeiro, Rio de Janeiro, Brazil; 3 Biological Mass Spectrometry Laboratory, The Scripps Research Institute, La Jolla, California, USA

## Abstract

**Background:**

A goal of proteomics is to distinguish between states of a biological system by identifying protein expression differences. Liu *et al*. demonstrated a method to perform semi-relative protein quantitation in shotgun proteomics data by correlating the number of tandem mass spectra obtained for each protein, or "spectral count", with its abundance in a mixture; however, two issues have remained open: how to normalize spectral counting data and how to efficiently pinpoint differences between profiles. Moreover, Chen *et al*. recently showed how to increase the number of identified proteins in shotgun proteomics by analyzing samples with different MS-compatible detergents while performing proteolytic digestion. The latter introduced new challenges as seen from the data analysis perspective, since replicate readings are not acquired.

**Results:**

To address the open issues above, we present a program termed PatternLab for proteomics. This program implements existing strategies and adds two new methods to pinpoint differences in protein profiles. The first method, ACFold, addresses experiments with less than three replicates from each state or having assays acquired by different protocols as described by Chen *et al*. ACFold uses a combined criterion based on expression fold changes, the AC test, and the false-discovery rate, and can supply a "bird's-eye view" of differentially expressed proteins. The other method addresses experimental designs having multiple readings from each state and is referred to as nSVM (natural support vector machine) because of its roots in evolutionary computing and in statistical learning theory. Our observations suggest that nSVM's niche comprises projects that select a minimum set of proteins for classification purposes; for example, the development of an early detection kit for a given pathology. We demonstrate the effectiveness of each method on experimental data and confront them with existing strategies.

**Conclusion:**

PatternLab offers an easy and unified access to a variety of feature selection and normalization strategies, each having its own niche. Additionally, graphing tools are available to aid in the analysis of high throughput experimental data. PatternLab is available at .

## Background

A goal of proteomics is to distinguish between states of a biological system by identifying protein expression differences [1]. Shotgun proteomics is a large-scale strategy for protein identification in complex mixtures that involves pre-digestion of intact proteins followed by peptide separation, fragmentation in a mass spectrometer, and database search. Its name is derived from DNA shotgun sequencing, which in turn follows the analogy of a shotgun's quasi-random firing pattern and dispersion to ensure the target is hit.

**Figure 1:**
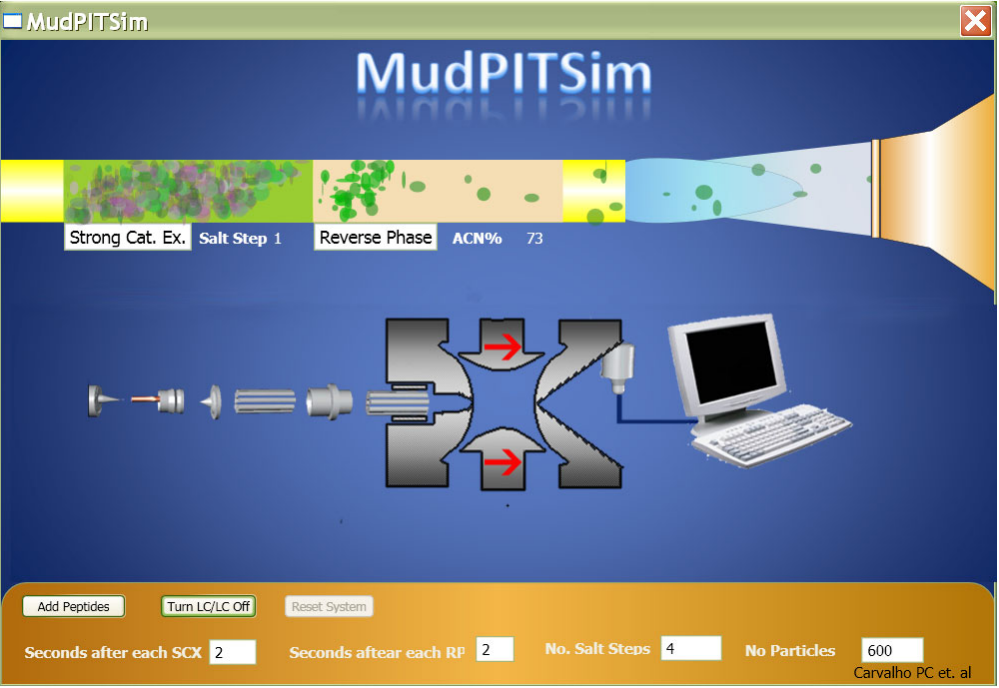
**MudPIT simulator**. The image displays the graphical user interface of the MudPIT simulator available on the project's website for didactic purposes. The simulator allows one to specify MudPIT parameters and then see the two-dimensional liquid chromatography simulation proceed on the fly. This is a simplification of reality; therefore, the timescale and many other features are not faithful representations. The green and pinkish structures in the upper part of the simulator represent the strong cation exchange and the reverse phase material packed in the capillary (yellow structure). The semi-conical structure represents the mass spectrometer nozzle (entrance) and the structure below is an X-Ray of a quadrupole ion trap.

Multi-dimensional Protein Identification Technology (MudPIT) is a shotgun proteomics technique capable of identifying thousands of proteins in proteolytically digested complex mixtures [2,3]. MudPIT separates peptides according to two independent physicochemical properties using two-dimensional liquid chromatography (LC/LC) online with the ion source of a mass spectrometer. This separation relies on columns of strong cation exchange (SCX) and reversed phase (RP) material, back to back, inside fused silica capillaries. The chromatography proceeds in cycles, each of which consists of increasing salt concentration to "bump" peptides off the SCX followed by a hydrophobic gradient to progressively elute peptides from the RP into the ion source. This process identifies mixture components by tandem mass spectrometry (MS/MS). For didactic purposes, a simplified and interactive MudPIT simulator is available at the project's web site; its interface is described in Figure 1.

Computational approaches for LC/MS-based differential proteomics usually involve *in silico *chromatogram alignment followed by pattern recognition strategies [4]. However, because of the more complex nature of MudPIT's LC/LC method and the alternating acquisition of mass spectra and tandem mass spectra, chromatographic alignment is more complicated than for LC/MS data. A milestone that eventually allowed differential MudPIT analysis was set with the development of alternative protein quantitation methods that use features from the tandem mass spectra (e.g., peptide hits, protein sequence coverage, spectral counts) as surrogate measures of protein abundance [2,5-7]. An important step was taken when Liu *et al*. demonstrated that the number of tandem mass spectra obtained for each protein, or "spectral count", correlates linearly with protein abundance in a mixture for two orders of magnitude [8]. These advances allowed LC/LC/MS/MS to produce semi-quantitative data on mixtures; however, two issues have remained open: how to normalize spectral count data for profile comparisons and how to statistically identify *bona fide *differences between samples (feature selection). Heretofore, differential proteomics by MudPIT spectral counting has relied on repeating assays to increase the number of identified proteins, improve protein coverage, and enable traditional statistical methods to pinpoint differences between biological states. Studies have shown Student's t-test, Fisher's exact test, and the G-test to be trustworthy for composing putative differential marker tallies when three or more replicates are available [9].

Recently, Chen *et al*. increased peptide and protein identifications in complex protein mixtures by re-analyzing samples digested in the presence of different MS-compatible detergents [10]. Moreover, the improved proteolytic digestion protocols potentially increased identification of less abundant proteins. However, the experimental design described by Chen *et al*. introduced additional data analysis challenges, since replicate readings are not acquired.

The contributions by Liu *et al*. and Chen *et al*. serve as foundations for this work. Here, we introduce a simple to use, yet efficient and panoptic, software for differential shotgun proteomics that addresses the data analysis issues of the experimental designs mentioned above. Our software, PatternLab for proteomics, or just PatternLab as referred to throughout, achieves its goal by featuring two new data analysis methods in addition to other widely adopted statistical approaches. The first method, ACFold, addresses experiments with less than three replicates from each state (class) or having data acquired by different protocols, as described by Chen *et al*. ACFold uses a combined criterion based on expression fold changes and the AC test [11]; its importance is demonstrated here with experimental data. The other method addresses experimental designs that comprise multiple replicates from each state and is referred to as nSVM (natural support vector machine) because of its roots in evolutionary computing and statistical learning theory [12]. We benchmarked nSVM against the widely adopted Student's t-test over a spiked marker dataset and identified its niche. A detailed description of ACFold, nSVM, and PatternLab's overall architecture is given, and critical issues of each method and how they were addressed are provided in the Implementation section.

## Implementation

PatternLab's current version is optimized for LC/LC/MS/MS data using spectral counts. Its architecture comprises four core modules (parsing MS data, data normalization, feature selection, and analysis). These modules can be operated programmatically or through the graphical user interface (GUI) that also provides specialized graphing tools to aid interpretation. Details of each module and a walkthrough of PatternLab, including the two new feature selection procedures ACFold and nSVM, are described below.

### Parser module

Let "project" refer to one's experimental data from all MudPIT assays of all biological samples from both control and case states. PatternLab relies on the parser module to translate a project's MS data into an index file and a sparse matrix file. The index file lists all identified proteins within the project and assigns each one a unique Protein IDentification (PID) integer. As for the sparse matrix, each row follows the schema: ⟨class label⟩⟨PID⟩:⟨value⟩...⟨PID⟩:⟨value⟩. In the latter, ⟨class label⟩ ∈ {-1, +1} is used to identify a biological state (e.g., +1 for control and -1 for case); ⟨PID⟩ and ⟨value⟩ correspond, respectively, to a protein identification index in the project's index file and to the spectral count verified for that protein during the corresponding MudPIT analysis. So, for example, the row "+1 1:3 2:5 3:6" specifies an analysis from the positive class having spectral count values of 3, 5, and 6 for PIDs 1, 2, and 3, respectively, all other PIDs having value 0.

There are various softwares that identify proteins by matching tandem mass spectra according to a database of peptide sequences, such as SEQUEST [13] and MASCOT [14]. The current parser can address both SEQUEST followed by DTASelect [15] and MASCOT having results exported to the DTASelect format. To use the parser, one should place the DTASelect results from the control and case analyses in different folders and then simply indicate their paths in the GUI.

### Normalization module

One or more normalization methods can be applied to the sparse matrix. PatternLab currently implements: ln (natural logarithm), Z [16], Total Signal, Maximum Signal, and Row Sigma. The ln normalization is obtained by taking the natural logarithm of every value and aims at increasing the signal of the PIDs with low spectral counts with respect to the more abundant PIDs. The Z normalization is achieved by subtracting from each original value the mean of all values of the corresponding PID and dividing the result by the standard deviation of all values from the same PID; the mean then becomes 0 and the standard deviation 1. The Total Signal normalization is achieved by dividing each value by the sum of all values in the respective row. The Maximum Signal normalization is obtained by dividing each value by the largest value in its row; an underlying assumption is that, in each MudPIT analysis, peptide identifications were obtained at or near the capacity of the tandem MS instrument. The Row Sigma normalization is achieved by calculating the mean and standard deviation of all values in a row and then dividing each value by the mean plus three standard deviations. The latter is introduced in this work as a variation of the Maximum Signal normalization that better handles assays that obtained an exceedingly high maximum value for a protein; further advantages are addressed in the Results and discussion section.

### ACFold feature selection

The ACFold analysis introduced in this paper is intended to evaluate data from projects having less than three replicate assays per class or assays obtained using different mass spectrometry protocols as described by Chen *et al*. [10]. The ACFold analysis takes advantage of two accepted criteria in proteomics to pinpoint differences between samples: the generalized AC Test [11] and expression fold changes [9]. The algorithm first parses the project's data as described in the Parser module section. The sparse matrix is then compressed into two rows, one representing each class. The new rows' values for each PID are obtained by averaging the original values of the corresponding PIDs within their classes. But given the nature of the experiment at hand, it is likely that low-probability events (such as PIDs that obtained a spectral count of 1 in only one assay of one of the two classes) will not always be observed; this would result in a calculated average of 0 and imply a probability of 0 that is not justifiable by evidence according to Cromwell's rule. To avoid the zero-frequency problem [17] and make fold-change calculations possible, a pseudo spectral count of 1 is then added to each PID value of the two resulting rows, including the unobserved PIDs, following the process known as Laplace's rule of succession. PatternLab then calculates the AC test probabilities and the expression fold changes according to one of the user-specified normalization methods: Total Signal, Row Sigma, or None.

Finally, a false-discovery rate (FDR) is estimated by the Benjamini-Hochberg procedure [18] for a given fold-change cutoff. Let *m *be the number of identified proteins minus the number of proteins that failed to pass the fold-change cutoff test. For *1 *≤ *i *≤ *m*, let $H_{i}^{0}$ be the (null) hypothesis that the *i*th protein is not differentially expressed, and *p*_*i *_its *p*-value. Assuming *p*_1 _≤ *p*_2 _≤ ... ≤ *p*_*m *_(ties are broken so that no lower-fold protein ranks ahead of another having the same *p*-value), and α the minimum FDR at which a test can be called significant, let

$$k=\max\left\{i:p_{i}\leq \frac{i}{m}\alpha \right\}.$$

The null hypotheses $H_{k}^{0}$ are then rejected (i.e., the corresponding proteins are declared differentially expressed). If no such *i *exists, no hypothesis is rejected.

**Figure 2:**
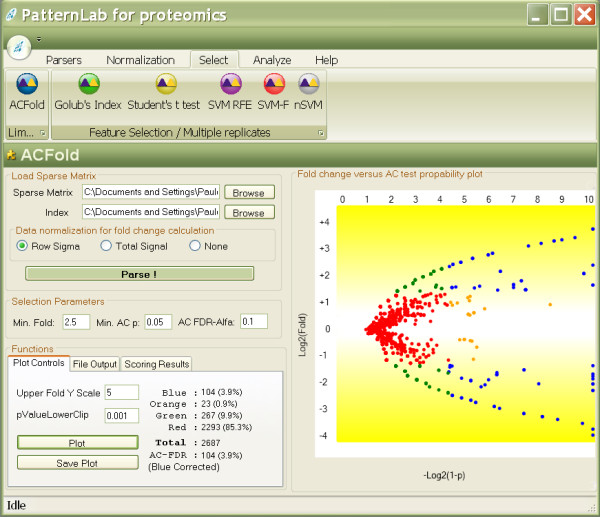
**ACFold's graphical user interface**. The interface above displays results from real experimental data. The plot on the right shows the distribution of the identified proteins according to log_2_(fold change) on the ordinate (y) and – log_2_(1- (AC test *p*-value)) on the abscissa (x). The plot tab indicates that 104 proteins (blue dots) were differentially expressed because they satisfied both the AC test and fold-change cutoffs specified by the user. 23 proteins (orange dots) did not meet the fold-change cutoff but were indicated as statistically differentially expressed, therefore deserving a second look. 267 proteins (green dots) met the fold-change cutoff; however, the AC test indicated that this happened by chance. 2293 proteins (red dots) were pinpointed as not differentially expressed between classes because they failed both the AC test and the fold-change cutoffs. The GUI also lists an AC FDR indicating that all blue dots satisfy the established user-selected FDR of 0.1.

The user can define stringency levels aided by a distribution plot and supplementary information offered by the GUI indicators. The stringency is performed by specifying a minimum fold-change cutoff, an AC test *p*-value cutoff, and the FDR α. We refer to Figure 2 to demonstrate how the results are presented. Lastly, the final report can be exported to text.

### nSVM feature selection

nSVM is a feature selection algorithm introduced in this work and used here to pinpoint differences in protein expression profiles when multiple replicates of each class are available. The algorithm begins by parsing the project's data as described in the Parser module section. nSVM then uses the structural risk minimization (SRM) principle from statistical learning theory [12] to drive the convergence of a genetic algorithm (GA). Briefly, a GA is a stochastic optimization technique inspired in evolutionary biology which imitates inheritance, selection, crossover, and mutation to evolve a population of abstract genomes (individuals) [19]. Each individual represents a candidate solution (set of differentially expressed PIDs) and is coded as an array of bits (1 or 0); the *n*th bit value hypothesizes that either the protein whose PID value is *n *is differentially expressed (1) or not (0). The general aim of a GA is to evolve an initial population of randomly generated individuals so that, after a number of generations, the solution will be encoded in the genome of the historically fittest individual.

The GA works by generating successive populations on the premise that the average individual fitness (quality of the solution) will increase for each new population. Each new solution from nSVM is produced by first selecting parents according to their quadratically normalized fitnesses. Formally, let *S *denote the set {*i*_0_, *i*_2_, ..., *i*_*n*-1_} of individuals, ordered by nondecreasing fitness. Let *j *and *k *be two randomly chosen numbers in the range from 0 to *n*^2^- 1. The two individuals chosen to mate will be the ones in *S *indexed by the greatest integers no greater than the square roots of *j *and *k *(i.e., the square roots' "floors"); if the same individual is chosen twice, this process is repeated. Clearly, fitter individuals have significantly higher chances of being selected. During the mating process, a uniformly random crossover operator is used, so the single offspring receives each gene (bit value) from either one of its parents with equal chances. The GA then performs mutations on the newly produced offspring according to a user-specified mutation index. For example, a mutation index of 2 allows the GA to perform up to two mutations in the offspring's genome. The mutation is performed by switching the values of randomly chosen bits with a bias towards mutating them to 0 (specifically, a 60% chance for 0 and 40% for 1). We recall that 0 and 1 represent excluding or including a feature, respectively. This bias accelerates the GA in finding solutions with fewer features. In addition, a fine-tuning parameter termed mutInd1After can be set through the GUI. This parameter stands for "Mutation Index 1 After", so after the algorithm has reduced the initial set of candidate proteins to a number below the one the parameter specifies, the mutation index is reduced to 1. This allows the GA to search within the remaining combinations with a lower probability of making great shifts away from the local optimum it is approaching. The processes of mating, crossover, and mutation are repeated until a population of the same size as the initial one is formed for use in the next iteration of the algorithm. The user can also configure the GA to allow "elitism", permitting a specified fraction of the fittest individuals to continue on to the new population. The algorithm terminates when a user-specified number of generations has elapsed without the appearance of an individual that is fitter than the fittest found so far.

Fitness evaluation is certainly one of the most important aspects of a GA. As far as we know, this is the first time a GA takes advantage of the SRM principle [12] to drive its convergence. Briefly, the SRM principle allows the evaluation of how well data points are separated in a feature space by a classification function, according to an empirical error measure on known examples and an upper bound on the function's error when generalizing for unknown examples [12]. The SRM principle is the basis of the SVM pattern recognition method, which searches for a classification function with the "best" trade-off between empirical error and worst-case generalization error. The upper bound on the generalization error grows monotonically with the machine's so-called VC dimension, so lower VC dimensions are preferred. Additionally, another upper bound on the generalization error depends on the machine's number of support vectors in a way that a small number of such vectors is also preferred [12]. We use this other bound as well. In the remainder of this section we refer to each row of the sparse matrix as an input vector.

**Figure 3:**
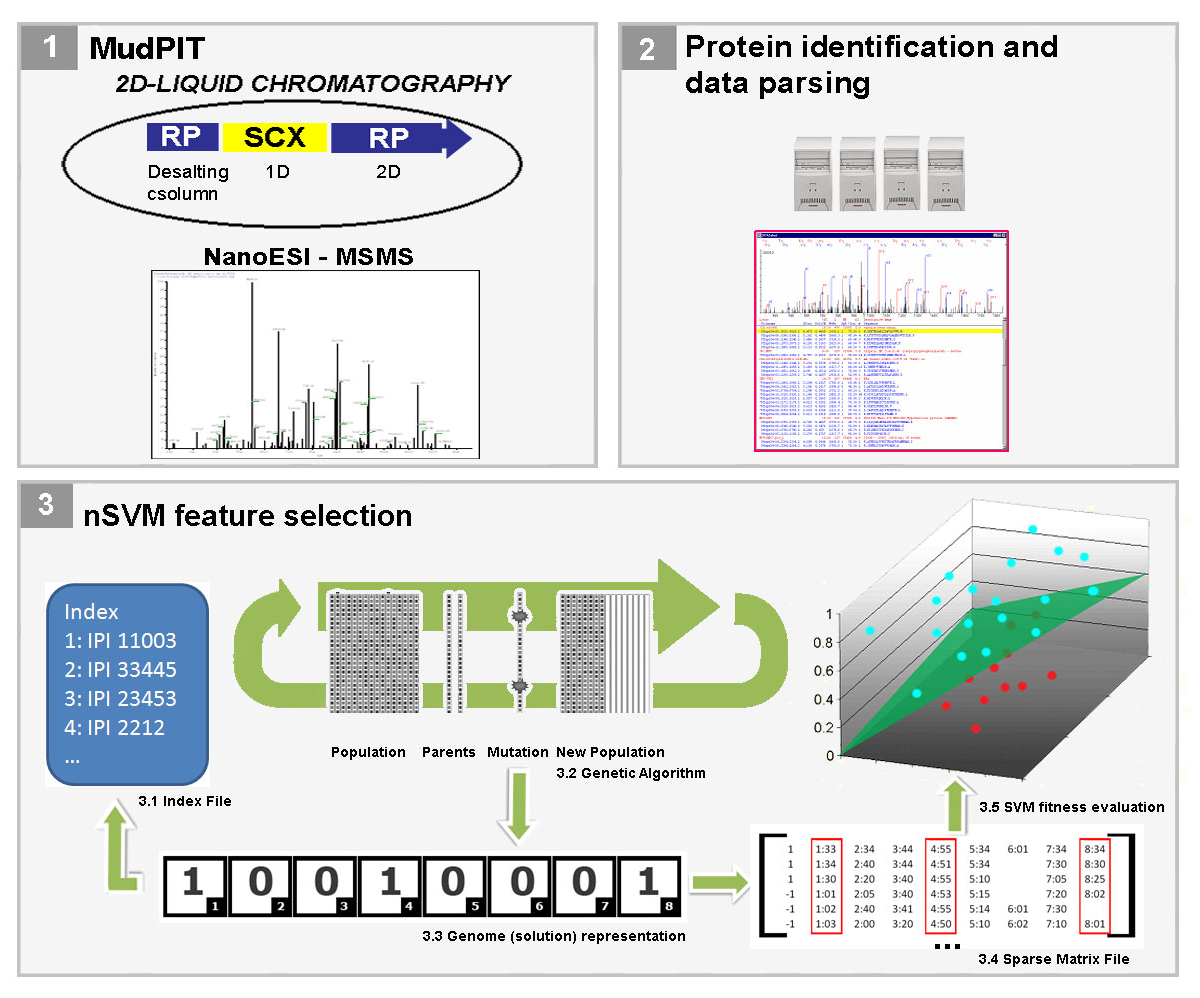
**nSVM's workflow**. MudPIT is applied to acquire mass spectrometry data from a biological system in different states (1). The data are subsequently identified by SEQUEST and filtered by DTASelect (2). nSVM is applied to pinpoint differences in the protein expression profiles by using a GA (3.2). Each individual's genome is an array of bits (3.3) that corresponds to a set of proteins (3.1 and 3.2) that will be selected from the dataset (3.4) to be evaluated as a solution (3.5) according to their spectral counts.

Each individual's fitness is evaluated by how well the input vectors are "separated" in the feature space defined by the individual. First, the input vectors are mapped onto the feature space taking into consideration only the proteins whose PIDs have value 1 in the individual. Secondly, an SVM model is generated and the empirical error is evaluated by the leave-one-out approach [20]. The VC dimension, the number of support vectors, and the number of bits having value 1 in the individual are also recorded. Finally, the fitness score for an individual is calculated as

$$fitnessScore=C_{1}LOO+C_{2}\left(1-\frac{1}{h}\right)+C_{3}\left(1-\frac{1}{nSV}\right)+C_{4}nG$$

where *LOO *is the SVM leave-one-out error, *h *is the VC dimension, *nSV *is the number of support vectors, *nG *is the number of bits with value equal to 1, and *C*_1 _through *C*_4 _are user-specified constants having default values set to 100, 100, 10, and 0.1, respectively. Clearly, the lower the score, the fitter the individual. We note that the first three parameters are calculated using SVM *light *[21]. Figure 3 summarizes the nSVM process up to this point.

nSVM relies on the island model to keep population diversity and to better address the issue of a large search space. This approach works with a user-specified number of populations that evolve independently. Individuals will migrate, from time to time, according to a user-specified time parameter. The migration proceeds as follows. First the GA randomly chooses two populations from its pool and pauses their computations after the fitness evaluation step. Secondly, a random number is picked (conforming to a user-specified upper bound) to indicate the number of individuals to be exchanged between the two populations. Thirdly, individuals are selected (as for mating, described above) and are exchanged between the populations. Finally, the GA continues to evolve both populations from where they were stopped. PatternLab takes advantage of the recent multi-core processors by having each population "live" in a different computing thread. Thus, a computer with a certain number of cores can manage as many populations concurrently without sacrificing performance.

The features for the final classification model are selected by executing nSVM multiple times (e.g., 20). For every nSVM execution, each time the fittest individual is replaced its genomic information is saved in a text file (history file). After multiple nSVM executions, several history files are available and a ranking of the features can be established according to the frequency of occurrence of each PID in the history files. Furthermore, a number of minimal discriminative features can be estimated by generating a two-column list having PIDs ordered by their ranks in the first column and their achieved frequencies in the second. The set of discriminative features is then estimated by locating, in this list, the two consecutive rows that present the greatest difference in frequency values. The number of features is then computed by counting how many features have scores above or equal to this gap's upper limit.

### Other available statistical inference methods and the result analyzer module

**Figure 4:**
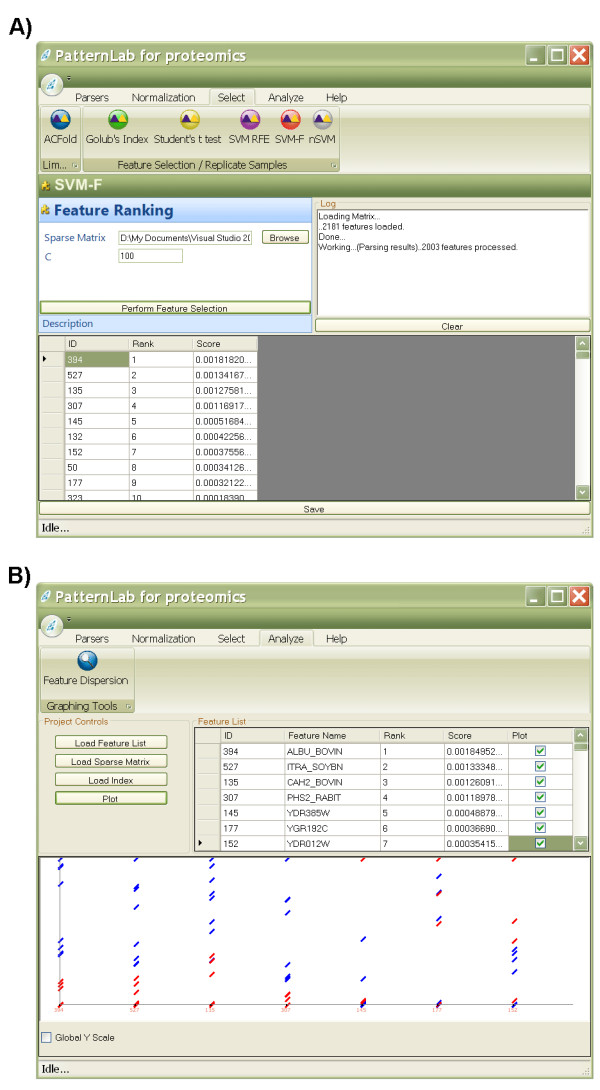
**Replicate experiment analyzer's graphical user interface**. This graphical user interface offers various normalization and feature selection methods (A). After applying the methods, the user can view the features ranked according to their scores. The expression from the selected feature can be graphed in the result analyzer (B).

**Figure 5:**
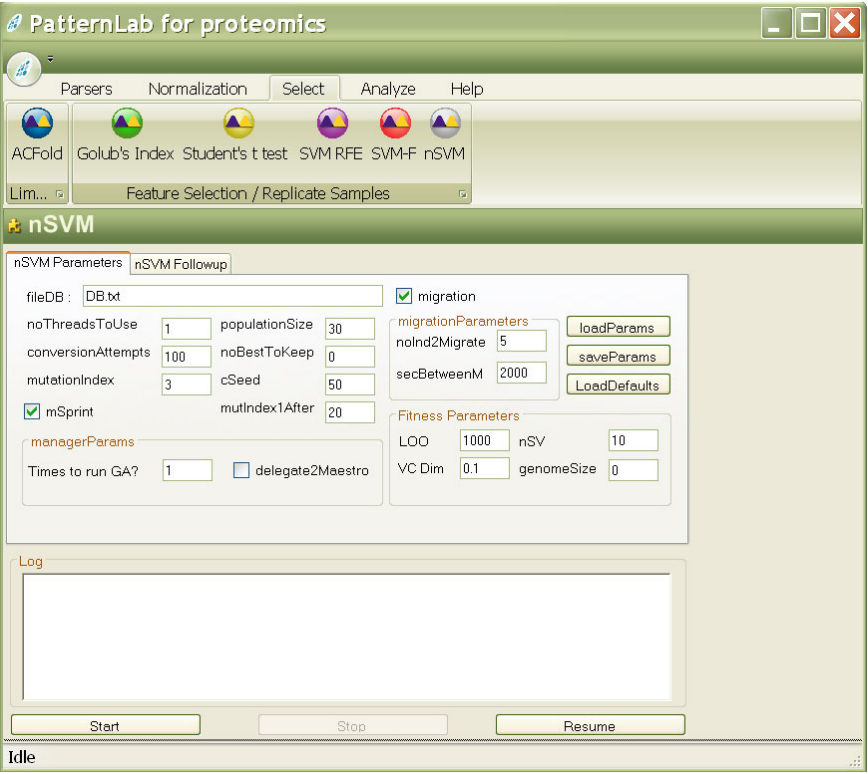
**nSVM's graphical user interface**. Every aspect of nSVM's GA can be customized in its graphical user interface or programmatically. A detailed explanation of each parameter can be obtained at the project's website.

PatternLab offers several additional feature selection methods that are widely adopted by the proteomics community. These methods include: SVM recursive feature elimination (SVM-RFE) [22], forward SVM (the weighting used in the first step of SVM-RFE), Golub's index [23], and Student's t-test [11]. It is beyond the scope of this manuscript to detail these methods since they are well documented in the literature. Figure 4 exemplifies PatternLab's GUI to access the feature selection methods and a result analyzer. In a future version of PatternLab, we intend to add new components to the result analysis module. Figure 5 exemplifies nSVM's interface.

## Results and discussion

Two main issues characterize feature selection challenges in bioinformatics: the large input dimensionality and limitations in the dataset size. To deal with these problems, various feature selection techniques have been designed by experts from the machine learning and data mining fields. The philosophy behind PatternLab is that there is no single, universally optimal feature selection technique [24]; additionally, the existence of more than one subset of features that discriminates the data equally well [25] should be considered. We believe that each feature selection strategy has its own niche, so it is important to know its idiosyncrasies, when to effectively apply it, and also to be aware of its limitations. For example, while the output provided by univariate feature rankings can be more intuitively grasped because they analyze each feature independently, protein subgroups that could possibly interact can only be detected through multivariate techniques (but requiring far more effort).

### Row Sigma normalization

Methods with ease of interpretation tend to be more readily accepted, which is in line with the possibility of intuitive interpretation that is one of the goals of the Row Sigma normalization strategy introduced in this work. This strategy joins the robustness of the Total Signal normalization (by using a measure that considers the entire profile through the sum of all values) with the ease of interpretation of the Maximum Signal normalization (by characterizing the proteins as percentages of an estimated most abundant protein). This is achieved by dividing the expression value of each protein by the mean plus three times the standard deviation taken inside its row in the sparse matrix. When compared to the Maximum Signal normalization, which relates all proteins solely to the most abundant one, Row Sigma normalization is seen to help avoid misleading conclusions that might be reached should the most abundant protein have a large variance associated with it.

### Suggestion of when to apply ACFold

ACFold combines the AC test with fold changes to pinpoint differentially expressed proteins between classes; this is important because conclusions drawn only from fold changes could be equivocated. For example, suppose spectral counts of 3 (6) and 30 (60) were observed for protein x (y) during the control (case) assay. Both x and y have a twofold up-regulation from the control to the case assay, but it is much likelier that the fold change for protein x happened by chance. The conditional probability of finding a spectral count of *x*_2 _in biological state 2 given that a spectral count of *x*_1 _was found in biological state 1 can be estimated by the AC test.

The AC test outputs a *p*-value related to testing a single hypothesis. In large-scale proteomic strategies, such as MudPIT, thousands of hypotheses are tested simultaneously, requiring an appropriate error rate control instead of relying solely on *p*-values. The most well-known strategy to deal with multiple hypotheses is the Bonferroni, but it is in some ways too conservative and this has led to the proposal of new ones [18,26]. The solution we propose to this massive multiple-hypothesis test problem is to analyze the data from an FDR perspective instead of that of *p*-values. The FDR is defined as the expected proportion of false positives among the results declared significant [18]. For example, by specifying an FDR of 0.1, it is expected that no more than 10% of the results declared significant (*p*-value ≤ cutoff) be false positives. This control, however, cannot be obtained with the *p*-value alone.

Label-free shotgun proteomics currently uses a random sampling process to estimate the relative quantitation of thousands of proteins. For this reason, determining the true number of differentially expressed proteins, which would require precise quantitation instead, has remained an open challenge. Due to the lack of a training set with known answers, our approach relies on a theoretical FDR estimator to cope with imprecise quantitation. One strategy to evaluate the effectiveness of FDR approaches is to spike protein markers with known concentrations into complex protein mixtures (e.g., lysates) to perform real, but controlled, experiments, which are therefore verifiable. For example, Zhang *et al*. [9] compared replicate LC-MS/MS assays of the *S. cerevisiae *lysate plus six protein markers (accounting for 1.25% and 2.5%, thus twofold, of the total protein content in the different classes) and observed that the true FDR of the AC test, when comparing two assays, varied from ~1% to ~13%, depending on the marker. Furthermore, the authors concluded that Fisher's exact test, the *G*-test, and the AC test all "give reasonable false positive rates even with limited sampling numbers from a single replicate." In view of these results, our choice to rely on a theoretical estimator instead of physical measurements seems justified.

Figure 2 exemplifies the ACFold analysis on experimental data. The aim was to identify as many proteins as possible in a glioblastoma cell culture when exposed (or not) to a chemotherapeutic agent during 1.5 h [27]. To maximize protein identification, different MS-compatible detergents were used during each MudPIT assay as described by Chen *et al*. [10]. Experiments that do not acquire replicate readings such as the latter or that have very few readings for each class (one or two) fall within ACFold's niche. As shown in Figure 2, 2687 proteins were identified, of which 104 were pinpointed as being differentially expressed according to ACFold (using a minimum fold-change cutoff of 2.5, an AC test *p*-value of 0.05, and an FDR of 0.1). The fold-change cutoff was empirically selected to maximize the number of hypotheses approved by the FDR criterion. For example, by specifying a cutoff of 2.0, the number of differentially expressed proteins according to the AC test was raised to 105; however, because of a great increase in the number of hypotheses tested, the FDR indicated only 88 as differentially expressed. When a cutoff of 3.0 was specified, both the number of differentially expressed proteins according to the AC test and the FDR were reduced to 85. We note, finally, that the FDR estimation is conservative for massively-multiple hypothesis testing [28] and that a high stringency on false positives can imply an increase in the number of false negatives; whence the choice of our FDR of 0.1.

### Suggestion of when to apply nSVM

Our observations suggest that nSVM's niche comprises projects targeting the selection of a minimum set of proteins (features) that nevertheless allows the highest rate of correctness to be achieved on unseen samples in classification problems. This selection entails the solution of the difficult bioinformatics combinatorial problem of choosing one out of the 2^*n *^sets into which *n *identified proteins can be combined. Two widely adopted classes of methodologies to solve this problem are the filter and the wrapper approaches. Briefly, the filter approach relies on a probabilistic method to eliminate or rank features, similarly for example to our use of the t-test. However, according to Cover and Van Campenhout [29], no ordering of error probabilities is guaranteed to produce the optimal feature subset or subsets. Moreover, feature sets can be algorithm-dependent to achieve good results. On the other hand, wrapper methods handle the problem by relying on the classification algorithm during the feature selection process, but the algorithm becomes more prone to overfitting.

nSVM is a wrapper feature selection approach that couples a nature-inspired optimization technique (a GA) with the state-of-the-art classifier (an SVM) to address the overfitting problem. This hybrid approach is justifiable because, even though SVMs efficiently generalize on noisy datasets, they have no internal feature relevance evaluation; therefore, noisy features can degrade their performance. Consequently, feature selection plays a key role prior to SVM classification, especially for complex datasets as in shotgun proteomics. Our GA is a stochastic heuristic that deals with massive resampling to handle the idiosyncrasies of a dataset as related to a classifier to avoid overfitting. Additionally, the feature sets selected by our GA are optimized for classification by an SVM because our GA's fitness function considers the same principles that drive the SVM classifier (i.e., the empirical error, the VC dimension, and the number of support vectors). Accordingly, we showed that nSVM efficiently dealt with the overfitting problem on a high-dimensional and noisy dataset by correctly pinpointing the relevant features (spiked proteins) and outperforming the t-test filter approach, as described below.

We demonstrate nSVM's niche using data from a real (yeast lysate replicates), yet controlled, experiment (protein markers were spiked to simulate differences), which is therefore verifiable. The dataset was obtained from four aliquots of 400 μg of a soluble yeast total cell lysate that were mixed with Bio-Rad SDS-PAGE low range weight standards containing phosphorylase b (PHS2), serum albumin (ALB), carbonic anhydrase (CAH), and trypsin inhibitor (ITRA) at relative levels of 25%, 2.5%, 1.25%, and 0.25% of the final mixtures' total weight. Four MudPIT assays were acquired for each aliquot as described by Liu *et al*. [8]. Finally, we generated three sparse matrices to simulate three benchmarking scenarios; in the first scenario, the input vectors originating from the 25% protein spiking were labeled as belonging to the positive class and all the rest as to the negative class. On the second scenario, the 25% and the 2.5% input vectors were labeled as from the positive class and the rest as from the negative class. Finally, the third scenario labels the 25%, 2.5% and 1.25% input vectors as belonging to the positive class and the remaining 0.25% as belonging to the negative class.

Each sparse matrix was normalized according to the Z method and nSVM was applied to predict which and how many markers were spiked in the first matrix (scenario 1) using the linear SVM kernel and varying some parameters of the GA (Table 1). Almost all parameter combinations correctly top-ranked the spiked markers, and pinpointed how many markers were spiked in the lysate. The dataset used for testing originated from a 12 salt step MudPIT of a whole cell yeast lysate having more than 1800 identified proteins; this is far more complex than the average proteomic experiment. Therefore, more combinatorial possibilities were available, increasing computation time. Given these facts, nSVM is expected to perform faster in less complex studies (with fewer features). The island mode and mutation index play a key role in the GA; while apparently there are no great changes in execution time, runs using a mutation index of 2 with the island mode turned on yielded better results in our dataset. We then opted to use the island model and a mutation index of 2 to evaluate nSVM over the other two scenarios; the method correctly predicted which markers were spiked in both cases (Table 2).

**Table 1:** Evaluation of nSVM results on the spiking dataset using different parameters

Elitism	Mutation	Islands	No. Feat.	Avg. No. Subst.	Time	PHS2	ALB	CAH	ITRA
0	2	0	6	290	49 ± 9	1	3	5	4
0	2	250	4	380	50 ± 8	1	2	4	3
0	3	0	5	400	54 ± 6	2	1	3	5
0	3	250	4	415	51 ± 9	1	3	5	6
1	1	250	4	280	49 ± 7	4	1	2	3
1	2	0	6	360	56 ± 7	1	2	4	3
1	2	250	4	410	59 ± 3	1	3	4	2
1	3	0	5	416	54 ± 5	5	3	4	7
1	3	250	6	423	52 ± 7	1	6	3	2

nSVM was executed 20 times for each of the 8 specified conditions. PHS2, ALB, CAH, and ITRA stand for the spiked protein markers, and the numbers in the respective columns indicate the ranking. The Elitism column stands for how many individuals of the population were allowed to remain untouched for the following generation. The Islands column indicates how many seconds were required before a migration could even occur; a zero indicates that the island model was not applied. The No. Feat. column indicates what nSVM suggested as the minimum set of optimal features. The Avg. No. Subst. column indicates how many times the fittest individual was substituted. The Time column indicates the average time and standard deviation of 1 nSVM run. These results were obtained for scenario 1 as described in the Suggestion of when to apply nSVM section. Due to the stochastic nature of the method, results may vary.

**Table 2:** nSVM results in the spiking dataset (scenarios 2 and 3)

Scenario	PHS2	ALB	CAH	ITRA
2	4	1	3	2
3	2	1	3	4

PHS2, ALB, CAH, and ITRA stand for the spiked protein markers, and the numbers in the respective columns indicate the ranking according to nSVM.

The Z normalization was chosen because one of the steps for estimating the VC dimension, according to the SVM *light *algorithm [21], is based on approximating the radius of the smallest hyphersphere that encompasses all input vectors by the norm of the largest support vector. After applying the Z normalization, the new mean for each PID becomes 0 and the data points become "evenly" distributed around the origin; this makes the VC dimension estimate more accurate. However, if the changes between samples are expected to be minimum, applying nSVM on "unnormalized" data can also be considered.

The widely adopted Student's t-test was then applied to check if we could rank the spiked markers as the topmost in the three scenarios after Z and Total Signal normalization. These results are listed in Table 3. By comparing the results from nSVM (Tables 1 and 2) against the t-test results (Table 3), it can be noted that the latter was unable to correctly rank the markers for some scenarios (all markers should have rank at most 4) and therefore did not reveal the optimal set. On the other hand, nSVM correctly ranked the spiked markers from all sparse matrices, which justifies the extra computation time it required. Recall that nSVM encompasses multiple executions (e.g., 20), and therefore more time to terminate (~1 h on an Intel Core 2 duo at 2.1 GHz). The limitations of the t-test seem to be that it relies on estimates of the mean and variance that do not necessarily reflect the true values when only a few samples are available [30]. nSVM's stochastic nature, combined with the various executions, makes it less prone to overfitting, but we note that it was unable to obtain the global optimum in any of the three matrices with only a single execution.

**Table 3:** Student's t-test results for the spiking experiment

Normalization Method	PHS2	ALB	CAH	ITRA
	scenario 1	
Z	3	1	25	2
Total Signal	2	18	187	30
	scenario 2	
Z	10	1	2	3
Total Signal	1	2	4	3
	scenario 3	
Z	111	2	1	5
Total Signal	5	2	1	4

PHS2, ALB, CAH, and ITRA stand for the spiked protein markers, and the numbers in the respective columns indicate ranking according to Student's t-test applied to the sparse matrix normalized by Z or Total Signal normalization.

We recommend the t-test over nSVM for experiments where many changes are expected. Table 2 shows that even though the optimal result was not always achieved, very satisfactory results were obtained. Therefore, the t-test can offer a quick "bird's eye" view over changes throughout the entire experiment. On the other hand, nSVM works its way down to a minimum set of features, optimized for classification purposes, and therefore is probably not advisable for a holistic view.

Differently than traditional GAs, nSVM offers a new strategy to estimate which proteins are differentially expressed. Our approach is a variation of the one used by Li *et al*. [31] to select differentially intensified mass spectral peaks from Surface Enhanced Laser Desorption Ionization – Time Of Flight proteomic profiles. Briefly, the authors repeatedly executed their GA rooted in k-nearest neighbor, a non-parametric pattern recognition method, to obtain relatively small subsets of discriminative mass spectral peaks between classes of specimens. Then peak appearance frequencies in the solutions were calculated and the authors showed that the most frequently selected peaks were the most discriminative. The efficiency of the algorithm was then proven on a validation set. The heuristics behind nSVM are far more computationally expensive than the one described by Li *et al*., so multiple executions (e.g., 1000) would invalidate its applicability. However, nSVM adopts a strategy that allows it to converge to very satisfactory solutions within only a few runs (e.g., 20).

Prior attempts at performing feature selection based solely on some function of the VC dimension [32] have been reported. However, our GA is based on the SRM principle that combines such a function with empirical error measures. Furthermore, we take advantage of a second theoretical error bound related to the number of support vectors to make nSVM converge faster (data not shown). We compared nSVM's performance with and without computing the empirical error measures; the former achieved better results on our dataset (data not shown).

## Conclusion

The identification of trustworthy protein markers is not an easy task, since mass spectrometry based proteomics is still in development and spectral counting effectiveness can vary on the experimental setup, including mass spectrometry type and data-dependent analysis configuration. PatternLab implements several existing strategies and adds two new tools to the proteomic data analysis arsenal, each one having its own niche. Our results showed that even in simple scenarios, where the spiked concentrations can be considered relatively high, the data can still play tricks on well-founded feature selection methods. This is due to the dataset's high dimensionality, sparseness, and lack of a known *a priori *probability distribution. In even more realistic and complex scenarios, markers might be present in extremely low concentrations. Modification in the experimental designs to isolate sub-proteomes is a solution; however, these separations are many times not straightforward if protein content is to be disturbed only minimally. Therefore, even with all the advances in pattern recognition techniques, a set of *bona fide *markers requires experimental and computational validation in unseen samples to ensure the model is not a result of overfitting.

## Availability and requirements

• **Project name: **PatternLab for proteomics

• **Project home page: **

• **Operating system(s): **Windows XP or VISTA. PatternLab is expected soon to run under Linux and Macintosh, thanks to the Mono project [33].

• **Programming language: **C#

• **Other requirements: **.NET 3.5

• **License: **GNU

• **Any restrictions to use by non-academics: **license needed

## Authors' contributions

PCC coded the software and wrote the first draft of the manuscript under the guidance of VCB and JRY. EIC and JSGF generated the MudPIT experimental, helped test the software, and suggested the inclusion of important features in it. All authors read and approved the final manuscript and contributed to the development of the project's website.
